# Perioperative Determinants of Functional Outcome and Mortality After Mechanical Thrombectomy Under General Anesthesia

**DOI:** 10.3390/jcm15093332

**Published:** 2026-04-27

**Authors:** Chanatthee Kitsiripant, Soraya Kongkaew, Nalinee Kovitwanawong, Jatuporn Pakpirom, Jutamas Onjan

**Affiliations:** Department of Anesthesiology, Faculty of Medicine, Prince of Songkla University, Hat Yai 90110, Thailand

**Keywords:** acute ischemic stroke, mechanical thrombectomy, general anesthesia, perioperative management, functional outcomes

## Abstract

**Background/Objectives**: Despite high recanalization rates associated with mechanical thrombectomy (MT), disability and death remain possible for many patients. Baseline stroke severity and reperfusion status predict outcomes; however, the influence of modifiable perioperative factors during general anesthesia (GA) remains unclear. We investigated actionable perioperative determinants of functional outcomes and 90-day mortality following MT under GA. **Methods**: We retrospectively analyzed 166 patients with acute ischemic stroke who underwent emergency MT with GA over 10 years (2014–2024). Poor functional outcomes were defined as a 90-day modified Rankin Scale score of 3–6, with all-cause 90-day mortality as the secondary endpoint. Independent predictors were identified using multivariable logistic regression, and discrimination was assessed using receiver operating characteristic analysis. **Results**: At 90 days, 56.6% of patients had poor functional outcomes, and mortality was 24.1%. Independent predictors of poor outcomes included preoperative hyperglycemia ≥ 140 mg/dL, vasopressor requirement, incomplete reperfusion, prolonged ventilator duration, and severe post-procedural neurological deficit. Optimal anesthetic induction dosing was strongly protective. Shorter groin puncture-to-recanalization time predicted better functional recovery. Mortality was associated with hyperglycemia, National Institutes of Health Stroke Scale ≥ 16, poor reperfusion, and prolonged ventilation. The models demonstrated excellent discrimination (area under the curve, 0.879 for poor outcomes; 0.923 for mortality). Perioperative physiological factors remained associated with outcomes independent of procedural success. **Conclusions**: Beyond technical success, perioperative physiological stability strongly influenced outcomes following MT under GA. Optimization of metabolic control, hemodynamic stability, procedural efficiency, and early ventilator liberation represents a clinically actionable strategy for improving neurological recovery and survival.

## 1. Introduction

Mechanical thrombectomy (MT), the standard of care for acute ischemic stroke caused by large vessel occlusion, achieves high reperfusion rates and improved functional outcomes [1,2,3,4,5,6]. Despite the high recanalization rates, over half of patients remain functionally dependent [2]. This variability has been linked to various established prognostic factors, such as age, baseline stroke severity, infarct burden, and reperfusion status [7,8,9,10,11].

General anesthesia (GA) is frequently used during MT to ensure airway protection, patient immobility, and procedural efficiency. However, GA may introduce physiological perturbations, such as hypotension, impaired cerebral perfusion, hyperglycemia, and delays in workflow, which could exacerbate secondary brain injury. Prior studies comparing GA with conscious sedation have yielded conflicting results, often conflating anesthetic modalities with physiological management and limiting mechanistic interpretation [12,13,14,15].

Data on the role of modifiable perioperative anesthetic and physiological factors in determining the outcomes following MT are lacking. Identifying such factors may provide actionable targets to improve neurological recovery beyond technical recanalization alone. The relative contribution of perioperative physiological factors, independent of baseline severity and reperfusion success, remains poorly defined; therefore, we aimed to evaluate perioperative predictors of functional outcomes and mortality in patients undergoing MT under GA, with a focus on clinically modifiable determinants.

## 2. Methods

### 2.1. Study Design and Participants

This retrospective cohort study was conducted at the Prince of Songkla University Hospital following approval by the Human Research Ethics Committee (approval no. 67-044-8-1) and registration in the Thai Clinical Trials Registry (TCTR number: 20251209002). We identified the predictive factors of poor outcomes in patients with acute ischemic stroke who underwent emergency MT under GA.

This study included 166 consecutive adults who underwent emergency MT under GA for acute ischemic stroke between January 2014 and August 2024. Patients with incomplete post-procedural admission data or missing 90-day follow-up evaluations were excluded from the study.

### 2.2. Anesthetic Management

GA was administered according to institutional guidelines, with induction agents including propofol, etomidate, midazolam, or combinations thereof. Induction dosing was categorized as optimal or non-optimal based on institutional protocols. An optimal induction dose was defined as a weight-adjusted regimen tailored to the patient’s clinical condition. It aimed to provide sufficient anesthesia while maintaining hemodynamic stability without clinically significant hypotension. Meanwhile, non-optimal induction dosing was any regimen not meeting these criteria, including both underdosing (insufficient anesthetic depth) and overdosing (excessive hemodynamic depression), based on anesthetic records and clinical documentation.

Endotracheal intubation was performed prior to or during the procedure as clinically indicated. Maintenance of anesthesia was primarily achieved with volatile anesthetics or propofol infusion. Hemodynamic management was guided by standard monitoring, with vasopressors administered at the attending anesthesiologist’s discretion.

### 2.3. Sample Size Calculation

The required sample size was estimated using a two-tailed comparison of the mean duration of mean arterial pressure (MAP) reduction > 15 mmHg between the outcome groups, as previously described [16]. Preliminary values indicated mean durations of 57.8 min (standard deviation [SD] 56.7) in patients with favorable outcomes and 93.6 min (SD 63.4) in those with poor outcomes. Using an α of 0.01, 90% power, and a 1:1 allocation ratio, the calculated sample size was 83 per group, yielding a total of 166 participants.

### 2.4. Outcomes

The primary outcomes were functional disability, defined as a modified Rankin Scale (mRS) score of 3–6 at 90 days, and all-cause 90-day mortality. Secondary outcomes included successful reperfusion assessed using the Thrombolysis in Cerebral Infarction (TICI) score and major procedural complications, such as groin hematoma, cerebral vasospasm, vessel perforation, and hemorrhagic transformation.

### 2.5. Data Collection

Clinical, anesthetic, and procedural data were retrieved from electronic medical records and institutional surgical databases. Neurological outcomes were assessed at the 90-day follow-up using the mRS, with scores of 0–2 classified as favorable and 3–6 as poor outcomes. All data were recorded in a secure electronic database using anonymized patient identification codes. Personal identifiers were removed to ensure confidentiality.

### 2.6. Statistical Analysis

Statistical analyses were conducted using R version 4.4.1 (R Core Team, 2024) [17]. Continuous variables were compared using Student’s *t*-test or the Wilcoxon rank-sum test, whereas categorical variables were analyzed using Pearson’s chi-squared or Fisher’s exact test, as appropriate. Variables with *p* < 0.20 in univariate analyses were entered into a backward stepwise multivariable logistic regression model to identify independent predictors of poor outcomes, with statistical significance set at *p* < 0.05.

To lower the risk of potential overadjustment bias, sensitivity analyses were conducted by rerunning multivariable models that excluded post-procedural variables (post-procedural National Institutes of Health Stroke Scale [NIHSS], ventilator days, and hospital length of stay) and retained only preoperative and intraoperative variables. Multicollinearity was assessed using variance inflation factors (VIFs): VIFs < 5 were considered acceptable. Model calibration was evaluated using the Hosmer–Lemeshow goodness-of-fit test and calibration plots generated using bootstrap resampling. To reduce the risk of overfitting, events per variable (EPV) were calculated: EPV ≥ 10 was considered acceptable. The results of sensitivity analyses are provided in Supplementary Tables S3 and S4.

## 3. Results

### 3.1. Patient Characteristics and Procedural Profiles

**Figure 1:**
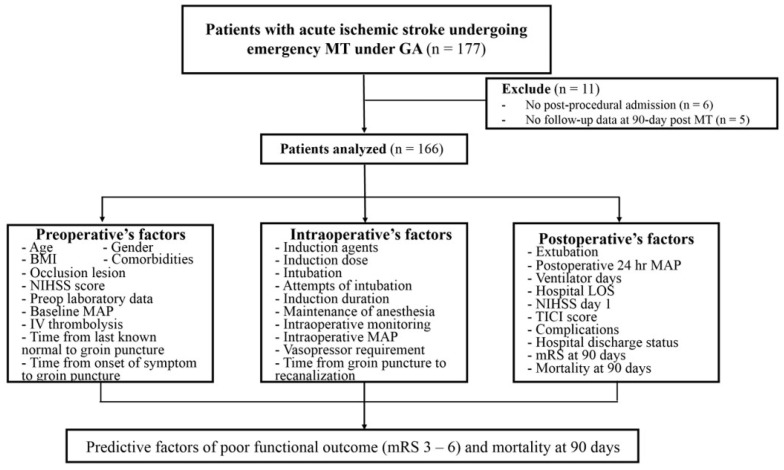
Study flow diagram and variable framework. Of the 177 patients who underwent emergency mechanical thrombectomy under general anesthesia, 11 were excluded (no post-procedural admission, *n* = 6; missing 90-day follow-up, *n* = 5), and 166 patients were included in the analysis. Preoperative, intraoperative, and postoperative variables were evaluated to identify predictors of poor functional outcomes (mRS 3–6) and 90-day mortality. BMI, body mass index; GA, general anesthesia; IV, intravenous; LOS, length of stay; MAP, mean arterial pressure; mRS, modified Rankin Scale; MT, mechanical thrombectomy; NIHSS, National Institutes of Health Stroke Scale; TICI, Thrombolysis in Cerebral Infarction.

A total of 166 patients undergoing emergency MT under GA were included in this analysis (Figure 1). Baseline characteristics are summarized in Table 1.

Patients presented with moderate-to-severe neurological deficits and a mean baseline NIHSS score of 14.4 ± 4.8; 39.1% had NIHSS ≥ 16. Anterior circulation stroke predominated. Approximately one-third of all patients demonstrated preoperative hyperglycemia (≥140 mg/dL), suggesting a substantial burden of metabolic derangement at presentation.

Patients with poor functional outcomes were significantly older (75.5 vs. 64.5 years, *p* < 0.001), had a higher prevalence of diabetes mellitus (35.1% vs. 13.9%, *p* = 0.004), and presented with more severe neurological deficits (NIHSS scores 15.5 ± 4.5 vs. 12.9 ± 4.8, *p* < 0.001). Furthermore, preoperative hyperglycemia (≥140 mg/dL) was more frequent in this group (47.9% vs. 25.0%, *p* = 0.004). Other baseline characteristics, including sex, body mass index (BMI), vascular comorbidities, anterior circulation involvement, and hematocrit levels, did not significantly differ between groups.

In mortality analysis, nonsurvivors had higher baseline NIHSS scores (16.1 ± 4.8 vs. 13.8 ± 4.6, *p* = 0.008), a greater prevalence of diabetes (40.0% vs. 21.4%, *p* = 0.033), and higher rates of preoperative hyperglycemia (60.0% vs. 31.0%, *p* = 0.002). Dyslipidemia was less frequent among nonsurvivors (30.0% vs. 51.6%, *p* = 0.028), while other baseline variables showed no association with mortality.

### 3.2. Perioperative and Procedural Factors

Table 2 summarizes perioperative and procedural characteristics. Optimal induction dosing was more frequent in patients with good functional outcomes (45.8% vs. 23.6%, *p* < 0.001), whereas vasopressor use was higher in the poor outcome group (71.3% vs. 50.0%, *p* = 0.008), suggesting greater perioperative hemodynamic instability. Other hemodynamic parameters, including arterial line monitoring and the magnitude or duration of MAP reduction, showed no association with outcomes.

Successful reperfusion was more commonly achieved in patients with good outcomes (91.7% vs. 76.6%, *p* = 0.018), whereas groin puncture-to-recanalization time showed a nonsignificant trend toward longer duration in the poor-outcome group (70.5 vs. 55.0 min, *p* = 0.078).

In mortality analysis, anesthetic variables, including induction dosing and vasopressor use, were not associated with mortality.

### 3.3. Post-Procedural Factors and Early Clinical Trajectory

Post-procedural variables demonstrated the strongest associations with outcomes. Severe neurological deficit following thrombectomy (NIHSS ≥ 16) was more frequent in patients with poor outcomes (27.7% vs. 1.4%, *p* < 0.001).

These patients also required longer mechanical ventilation (median 5 vs. 2 days, *p* < 0.001) and had a higher incidence of hemorrhagic transformation (51.1% vs. 23.6%, *p* < 0.001). Hospital length of stay did not significantly differ between groups.

In mortality analysis, nonsurvivors exhibited a similar but more pronounced pattern. Severe post-procedural neurological deficit was substantially more frequent (45.0% vs. 6.3%, *p* < 0.001), accompanied by lower rates of successful reperfusion (70.0% vs. 87.3%, *p* = 0.021) and longer ventilator duration (median 4 vs. 3 days, *p* = 0.014). Hospital stay was significantly shorter in nonsurvivors (median 5 vs. 10.5 days, *p* < 0.001), reflecting early mortality rather than improved recovery.

These post-procedural variables reflect downstream clinical consequences and could partially mediate the observed associations.

### 3.4. Independent Predictors of Poor Functional Outcome at 90 Days

Multivariable logistic regression analysis identified several independent predictors of poor functional outcomes at 90 days (Table 3). Preoperative hyperglycemia (≥140 mg/dL) significantly increased the risk of poor outcomes (adjusted odds ratio [aOR] 3.94, 95% confidence interval [CI] 1.32–13.07; *p* = 0.018). Optimal induction dosing was strongly protective (aOR 0.16, 95% CI 0.05–0.52; *p* = 0.004), whereas vasopressor requirement markedly increased the likelihood of poor recovery (aOR 7.72, 95% CI 2.32–32.05; *p* = 0.002), reflecting perioperative hemodynamic instability.

Reperfusion quality remained a major determinant, with suboptimal reperfusion being strongly associated with poor outcomes (aOR 21.03, 95% CI 3.55–178.88; *p* = 0.002). Shorter time from groin puncture to recanalization was associated with better functional recovery (aOR 0.99, 95% CI 0.98–1.00; *p* = 0.042). Severe post-procedural neurological deficit (NIHSS ≥ 16) was the most powerful predictor (aOR 191.15, 95% CI 14.38–8651.68; *p* < 0.001).

Prolonged ventilator duration was independently associated with worsened outcomes (aOR 1.38, 95% CI 1.19–1.68; *p* < 0.001). Conversely, a longer hospital stay was modestly inversely associated (aOR 0.96, 95% CI 0.93–0.99; *p* = 0.003), likely reflecting early mortality among critically ill patients.

### 3.5. Independent Predictors of 90-Day Mortality

The independent predictors of mortality are shown in Table 4. Preoperative hyperglycemia increased the risk of mortality (aOR 3.15, 95% CI 1.08–9.53; *p* = 0.037). Severe post-procedural neurological deficit (NIHSS ≥ 16) was the strongest determinant of death (aOR 43.87, 95% CI 5.22–1005.79; *p* = 0.002). Poor reperfusion independently predicted mortality (aOR 6.86, 95% CI 1.69–31.72; *p* = 0.009), as did prolonged ventilator duration (aOR 1.40, 95% CI 1.21–1.68; *p* < 0.001). A shorter hospital stay was associated with early death among nonsurvivors (aOR 0.74, 95% CI 0.63–0.84; *p* < 0.001).

### 3.6. Multivariable Model Performance

**Figure 2:**
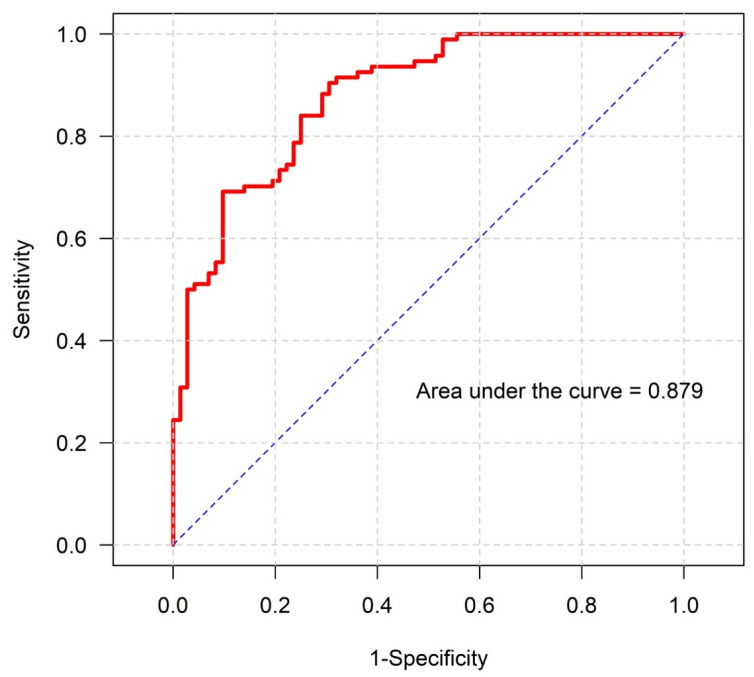
Receiver operating characteristic curve of the final multivariable model predicting poor functional outcome (modified Rankin Scale scores 3–6) at 90 days post-mechanical thrombectomy under general anesthesia. The model demonstrated excellent discrimination (area under the curve = 0.879).

**Figure 3:**
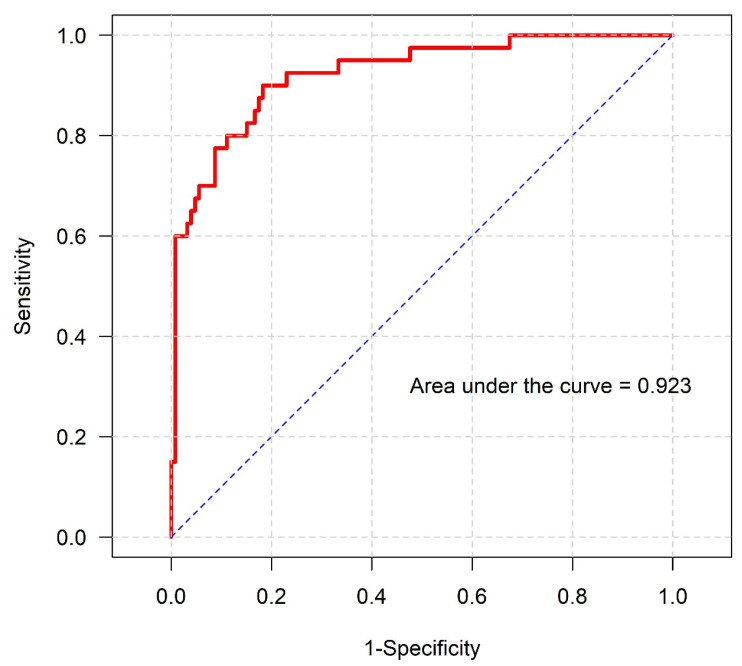
Receiver operating characteristic curve of the final multivariable model predicting 90-day mortality post-mechanical thrombectomy under general anesthesia. The model demonstrated excellent discrimination (area under the curve = 0.923).

The final multivariable model demonstrated excellent discriminative performance, with an area under the receiver operating characteristic curve (AUC) of 0.879 for predicting poor functional outcome (Figure 2) and 0.923 for predicting 90-day mortality (Figure 3). The sensitivity models demonstrated comparable discriminative performance (Supplementary Figures S1 and S2).

Sensitivity analyses that excluded post-procedural variables yielded consistent directional associations, although effect sizes were attenuated. This supports the robustness of the findings and suggests partial mediation through post-procedural neurological injury.

Multicollinearity assessment demonstrated low VIF values across all variables (mRS 90: 1.09–1.31; mortality 90: 1.00–1.25), suggesting no multicollinearity. Model calibration was acceptable, with non-significant Hosmer–Lemeshow test results (*p* > 0.05) and good agreement between predicted and observed probabilities (Supplementary Figures S3 and S4). EPV ratios were within acceptable ranges, supporting model stability and low risk of overfitting. Furthermore, comparable discriminative performance was observed in sensitivity models (Supplementary Figures S5 and S6).

## 4. Discussion

In this decade-long cohort study of patients with acute ischemic stroke undergoing MT under GA, we identified several perioperative physiological factors associated with functional outcomes and mortality at 90 days.

In addition to established determinants, such as baseline neurological severity and reperfusion success, modifiable perioperative variables—including hemodynamic instability, anesthetic management, and metabolic derangements—were independently associated with clinical outcomes. These findings highlight that, beyond technical success, perioperative physiological optimization represents a complementary therapeutic target in modern stroke care.

Preoperative hyperglycemia was independently associated with poor functional outcomes and mortality. Hyperglycemia exacerbates ischemic injury through oxidative stress, endothelial dysfunction, blood–brain barrier disruption, and thromboinflammatory microvascular failure [18,19], thereby reducing the salvageable penumbra even following successful recanalization. These findings support metabolic optimization as a clinically actionable perioperative target and reinforce the current neurocritical care recommendations advocating early glycemic control when glucose levels exceed 140 mg/dL in acute ischemic stroke [20].

Maintaining cerebral perfusion during thrombectomy is vital, especially in patients with impaired autoregulation and vulnerable penumbrae. In this group, the need for vasopressors, rather than absolute MAP reduction, was independently linked to poor outcomes, indicating that overall hemodynamic instability may be more important than reaching a specific hypotension threshold. Current neuroanesthesia guidelines recommend maintaining a systolic blood pressure > 140 mmHg [20], and prior studies have demonstrated a U-shaped relationship between blood pressure and stroke outcomes [21,22]. Intraprocedural MAP reductions > 40% from baseline are associated with unfavorable neurological recovery [23].

Herein, absolute MAP decline and hypotension duration were not independently predictive following adjustment, possibly reflecting rapid correction and tight hemodynamic control. Continuous arterial monitoring may reduce peri-induction hypotension [24], but can delay reperfusion [8,9], highlighting the need to balance monitoring precision with procedural efficiency. These findings support a physiology-guided strategy that prioritizes sustained cerebral perfusion stability over rigid adherence to isolated blood pressure thresholds. We recognize that reduced induction dosing might have been intentionally used in patients with greater clinical severity or hemodynamic vulnerability, corresponding to appropriate anesthetic adjustment rather than suboptimal practice. As such, non-optimal induction dosing may, in part, represent a marker of underlying disease severity rather than a purely causal factor.

Reperfusion quality remained a major determinant, consistent with rapid and effective recanalization being the strongest predictor of favorable neurological recovery [25,26]. In addition, a shorter groin puncture-to-recanalization time was independently associated with improved functional outcomes, reinforcing the importance of procedural efficiency in preserving viable brain tissue. Registry data suggest that the clinical benefit of first-pass recanalization is largely driven by faster reperfusion rather than by completeness alone [27,28,29], underscoring the critical role of procedural efficiency. However, technical success alone does not guarantee recovery.

Hemorrhagic transformation and post-reperfusion injury likely reflect microvascular damage and neuroinflammatory cascades, emphasizing the importance of optimal physiological control to translate successful recanalization into meaningful neurological recovery.

The post-procedural NIHSS score was the strongest predictor of disability and mortality, indicating that early neurological status reflects the integrated effects of baseline injury, reperfusion success, and perioperative physiology. In our cohort, a post-procedural NIHSS score of ≥16 was strongly associated with 90-day mortality, suggesting that substantial neurological injury might already be established despite technically successful recanalization. This finding is consistent with previous studies demonstrating early post-thrombectomy neurological severity as a key determinant of long-term functional outcomes and survival [6,7], supporting its value for early prognostication and risk stratification after MT.

Prolonged ventilator duration was also independently associated with poor functional outcomes and mortality [29], likely reflecting both neurological severity and systemic complications, such as impaired airway protection, pneumonia, and systemic inflammation. Previous studies have consistently shown that prolonged ventilation is associated with higher pneumonia rates, worse functional outcomes, and increased mortality, whereas a shorter ventilation duration and earlier safe extubation are associated with improved neurological recovery and survival [30,31].

The inverse association between the length of hospital stay and mortality should be interpreted with caution, as shorter hospitalization among nonsurvivors most likely reflects early death rather than a protective effect. Taken together, these findings indicate that neurological injury and systemic burden remain key determinants of long-term outcomes beyond procedural success alone.

The current study aimed to identify factors associated with outcomes, not to establish causality or predictive models. Therefore, the observed associations should be interpreted within this context. Several variables, such as vasopressor requirement and ventilator duration, may partly reflect disease severity rather than direct causes, consistent with confounding by indication. Sensitivity analyses excluding post-procedural variables demonstrated consistent directional associations with attenuated effect sizes, supporting the robustness of the findings and suggesting partial mediation through post-procedural neurological injury.

The study period spanned a decade, during which significant progress in thrombectomy techniques, operator experience, and perioperative management has occurred. In this study, no formal adjustment for temporal trends was performed; however, the core perioperative variables examined—such as hemodynamic management, anesthetic dosing, and metabolic control—represent fundamental physiological principles that are relatively consistent across clinical practice. As such, the observed associations are less likely to be solely attributed to temporal changes in procedural techniques. Nevertheless, unmeasured temporal variation may have influenced the results and should be considered when interpreting these findings.

The findings of this study have important clinical implications. The identification of modifiable perioperative physiological factors suggests that optimizing metabolic control, hemodynamic stability, anesthetic management, and procedural efficiency enhances outcomes beyond recanalization alone. These results support a shift from a purely procedure-centered approach toward a physiology-guided strategy in thrombectomy care. Future prospective studies incorporating standardized perioperative protocols and temporal stratification should validate these findings and further define causal relationships.

This study has some limitations. First, its retrospective, single-center design may limit generalizability and introduce residual confounding. Second, despite adjusting for baseline severity and additional sensitivity analyses, confounding by indication cannot be fully excluded. Third, post-procedural variables are potential mediators rather than independent predictors; their inclusion in primary models may influence effect estimates. Finally, temporal changes in practice patterns were not formally modeled.

## 5. Conclusions

In patients undergoing MT under GA, perioperative physiological stability is strongly associated with functional outcomes and mortality beyond technical success alone. Optimizing metabolic control, hemodynamic stability, procedural efficiency, and early ventilator liberation improves neurological recovery and survival. The findings of this study support a shift from a purely procedure-centered approach toward a physiology-guided strategy in thrombectomy care.

## Figures and Tables

**Table 1:** Baseline demographic and stroke characteristics stratified by 90-day functional outcome and mortality.

Variable	Good Outcome (mRS 0–2) (n = 72)	Poor Outcome (mRS 3–6) (n = 94)	*p*-Value	Survivors (n = 126)	Nonsurvivors (n = 40)	*p*-Value
**Demographics**						
Age (years), median (IQR)	64.5 (55.8, 74)	75.5 (63.2, 80.8)	<0.001	70.5 (61, 78)	74.5 (57, 78.2)	0.813
Sex			0.06			0.530
Male	51 (70.8)	52 (55.3)		76 (60.3)	27 (67.5)	
Female	21 (29.2)	42 (44.7)		50 (39.7)	13 (32.5)	
BMI (kg/m^2^), median (IQR)	23.4 (21.5, 25.8)	23.6 (20.8, 26)	0.794	23.5 (21.3, 25.9)	23.4 (20.3, 25.7)	0.565
**Comorbidities**						
Diabetes mellitus	10 (13.9)	33 (35.1)	0.004	27 (21.4)	16 (40)	0.033
Hypertension	40 (55.6)	63 (67)	0.178	78 (61.9)	25 (62.5)	1
Dyslipidemia	34 (47.2)	43 (45.7)	0.974	65 (51.6)	12 (30)	0.028
Prior stroke	18 (25)	22 (23.4)	0.956	28 (22.2)	12 (30)	0.430
Atrial fibrillation	27 (37.5)	46 (48.9)	0.189	58 (46)	15 (37.5)	0.445
**Stroke characteristics**						
Preoperative NIHSS, Mean ± SD	12.9 ± 4.8	15.5 ± 4.5	<0.001	13.8 ± 4.6	16.1 ± 4.8	0.008
NHISS ≥ 16	21 (29.2)	44 (46.8)	0.059	45 (35.7)	20 (50)	0.091
Anterior circulation stroke	66 (91.7)	79 (84.0)	0.501	94 (74.6)	31 (77.5)	0.379
Prior treatment with IV thrombolysis	39 (54.2)	44 (46.8)	0.434	64 (50.8)	19 (47.5)	0.856
**Laboratory variables**						
Preoperative glucose ≥ 140 mg/dL	18 (25)	45 (47.9)	0.004	39 (31.0)	24 (60.0)	0.002
Hematocrit (%), median (IQR)	40.3 (36.7, 43.2)	39.1 (35.1, 41.9)	0.116	39.9 (36.2, 42.6)	37 (34.6, 41.9)	0.111

Data are presented as numbers (%) unless otherwise indicated. SD, standard deviation; BMI, body mass index; NIHSS, National Institutes of Health Stroke Scale; MAP, mean arterial pressure; IV, intravenous; IQR, interquartile range.

**Table 2:** Perioperative and procedural characteristics stratified by functional outcome and 90-day mortality.

Variable	Good Outcome (mRS 0–2) (n = 72)	Poor Outcome (mRS 3–6) (n = 94)	*p*-Value	Survivors (n = 126)	Nonsurvivors (n = 40)	*p*-Value
**Anesthetic and hemodynamic variables**						
Optimal induction dose	33 (45.8)	21 (23.6)	<0.001	47 (38.5)	9 (25.7)	0.232
Arterial line monitoring	31 (43.1)	54 (57.4)	0.093	64 (50.8)	17 (42.5)	0.464
Greatest MAP reduction (mmHg), mean ± SD	40.4 ± 17.1	42.7 ± 18.5	0.416	42.7 ± 17.8	38.7 ± 17.9	0.220
Duration of MAP reduction ≥ 30 mmHg (min), median (IQR)	42.5 (0, 72.5)	50 (5, 115)	0.132	65 (27.5, 100)	100 (50, 123.8)	0.073
Vasopressor use	36 (50)	67 (71.3)	0.008	77 (61.1)	26 (65)	0.799
**Procedural variables**						
Groin puncture to recanalization (min), median (IQR)	55 (37.5, 85)	70.5 (39.2, 111)	0.078	61 (38, 88.2)	75 (48.8, 114.8)	0.089
Successful reperfusion (mTICI 2b—3)	66 (91.7)	72 (76.6)	0.018	110 (87.3)	28 (70)	0.021
**Post-procedural variables**						
Post-procedural NIHSS ≥ 16,	1 (1.4)	26 (27.7)	<0.001	8 (6.3)	18 (45.0)	<0.001
Ventilator days (days), median (IQR)	2 (1, 3)	5 (3, 12)	<0.001	3 (2, 7)	4 (3, 7.8)	0.014
Hospital stay (days), median (IQR)	8 (5, 16)	12 (5, 20.8)	0.192	10.5 (6, 20.8)	5 (4, 14.5)	<0.001
**Complications**						
Hemorrhagic transformation	17 (23.6)	48 (51.1)	<0.001	45 (35.7)	20 (50)	0.154
**Outcomes**						
mRS at 90 days			<0.001			<0.001
0–2	72 (100)	1 (1.1)		73 (57.9)	0 (0)	
3–6	0 (0)	93 (98.9)		53 (42.1)	40 (100)	
Mortality at 90 days	0 (0)	40 (42.6)	<0.001			

Data are presented as numbers (%) unless otherwise indicated. IQR, interquartile range; NIHSS, National Institutes of Health Stroke Scale; SD, standard deviation; MAP, mean arterial pressure; IV, intravenous; mTICI, modified Thrombolysis in Cerebral Infarction; mRS, modified Rankin Scale.

**Table 3:** Multivariable logistic regression analysis of factors associated with poor functional outcome at 90 days.

Variable	Adjusted Odds Ratio	95% CI	*p*-Value
Hypertension	2.71	0.91–8.82	0.081
Hematocrit	0.94	0.86–1.01	0.102
Preoperative glucose ≥ 140 mg/dL	3.94	1.32–13.07	0.018
Optimal induction dose	0.16	0.05–0.52	0.004
Vasopressor use	7.72	2.32–32.05	0.002
Time from groin puncture to recanalization	0.99	0.98–1.00	0.042
Post-procedural NIHSS ≥ 16	191.15	14.38–8651.68	<0.001
Reperfusion grade (mTICI)	21.03	3.55–178.88	0.002
Ventilator days	1.38	1.19–1.68	<0.001
Hospital stay	0.96	0.93–0.99	0.003

CI, confidence interval; NIHSS, National Institutes of Health Stroke Scale; mTICI, modified Thrombolysis in Cerebral Infarction.

**Table 4:** Multivariable logistic regression analysis of factors associated with 90-day mortality.

Variable	Adjusted Odds Ratio	95% CI	*p*-Value
Hematocrit	0.95	0.88–1.03	0.225
Preoperative glucose ≥ 140 mg/dL	3.15	1.08–9.53	0.037
Post procedural NIHSS ≥ 16	43.87	5.22–1005.79	0.002
Reperfusion grade (mTICI)	6.86	1.69–31.72	0.009
Ventilator days	1.40	1.21–1.68	<0.001
Hospital stay	0.74	0.63–0.84	<0.001

CI, confidence interval; NIHSS, National Institutes of Health Stroke Scale; mTICI, modified Thrombolysis in Cerebral Infarction.

## Data Availability

All the data created or analyzed during this work are available from the corresponding author upon reasonable request.

## Supplementary Materials

The following supporting information can be downloaded at: https://www.mdpi.com/article/10.3390/jcm15093332/s1, Table S1: Baseline characteristics, perioperative variables, and outcomes of the enrolled participants; Table S2: Clinical, anesthetic, and procedural factors according to outcome and mortality at 90 days; Table S3: Multivariable logistic regression analysis for poor functional outcome (mRS 3–6) at 90 days after exclusion of post-procedural variables; Table S4: Multivariable logistic regression analysis for 90-day mortality after exclusion of post-procedural variables; Figure S1: Receiver operating characteristic curve of the sensitivity model for predicting poor functional outcome at 90 days after exclusion of post-procedural variables; Figure S2: Receiver operating characteristic curve of the sensitivity model for predicting 90-day mortality after exclusion of post-procedural variables; Figure S3: Calibration plot for the multivariable model predicting poor functional outcome at 90 days; Figure S4: Calibration plot for the multivariable model predicting 90-day mortality; Figure S5: Calibration plot of the sensitivity model for predicting poor functional outcome (mRS 3–6) at 90 days after exclusion of post-procedural variables; Figure S6: Calibration plot of the sensitivity model for predicting 90-day mortality after exclusion of post-procedural variables.

## Abbreviations

The following abbreviations are used in this manuscript:

aOR	adjusted odds ratio
AUC	area under the curve
CI	confidence interval
GA	general anesthesia
MAP	mean arterial pressure
mRS	modified Rankin Scale
MT	mechanical thrombectomy
mTICI	modified Thrombolysis in Cerebral Infarction
NIHSS	National Institutes of Health Stroke Scale
SD	standard deviation
TICI	Thrombolysis in Cerebral Infarction
