# Supplementary material for: Perioperative Determinants of Functional Outcome and Mortality After Mechanical Thrombectomy Under General Anesthesia
Source: J Clin Med. 2026 Apr 27;15(9):3332. doi: 10.3390/jcm15093332 (PMC13164159; doi:10.3390/jcm15093332)

Supplementary Table S1 Baseline characteristics, perioperative variables, and outcomes of the enrolled participants

Characteristics	Value
**Baseline demographic and clinical characteristics**	
Age (year), mean ± SD	68.5 ± 14.2
Sex	
Male	103 (62)
Female	63 (38)
BMI (kg/m^2), mean ± SD	23.5 ± 3.9
Comorbidities	
Diabetes mellitus	43 (25.9)
Hypertension	103 (62)
Dyslipidemia	77 (46.4)
Prior stroke	40 (24.1)
Atrial fibrillation	73 (44)
Coronary artery disease	33 (19.9)
Valvular heart disease	26 (15.7)
Malignancy	16 (9.6)
Smoking	30 (81.9)
**Stroke characteristics and preoperative variables**	
Occlusion location	
Internal carotid artery terminus	55 (33.1)
M1 segment	71 (42.8)
M2 segment	15 (9)
Vertebral artery	3 (1.8)
Basilar artery	18 (10.8)
Anterior communicating artery	2 (1.2)
A2 segment	1 (0.6)
Carotid artery	1 (0.6)
Preoperative NIHSS, mean ± SD	14.4 ± 4.8
1–4	4 (2.4)
5–15	97 (58.4)
16–20	51 (30.7)
21–42	14 (8.4)
Baseline MAP (mmHg), mean ± SD	109.8 ± 19
Prior treatment with IV thrombolysis	83 (50)
Time from last-known-well to groin puncture (min), mean ± SD	342.7 (196.2)
Time from onset to groin puncture (min), mean ± SD	286.6 (166.7)
Preoperative laboratory	
Hematocrit (%), mean ± SD	38.6 ± 6.6
Platelet (cell/mm^3), mean ± SD	237,475.9 ± 79,702.5
Glucose (mg/dL), mean ± SD	143.1 ± 51.4
Creatinine (mg/dL), mean ± SD	1.1 ± 0.8
**Anesthetic and intraoperative management**	
Induction agents	
Propofol	111 (66.9)
Etomidate	2 (1.2)
Midazolam	15 (9)

≥2 agents	29 (17.5)
None	9 (5.4)
Induction dose (n = 157)	
Optimal dose	66 (42)
Non-optimal dose	91 (58)
Intubation location	
Referring hospital	25 (15.1)
Emergency room	10 (6)
Ward	4 (2.4)
Interventional suite	127 (76.5)
Intubation attempt	
1 attempt	163 (98.2)
≥2 attempts	3 (1.8)
Induction duration (min), mean (IQR)	15 (10,20)
Maintenance of anesthesia	
Sevoflurane	160 (96.4)
Desflurane	5 (3)
Propofol infusion	1 (0.6)
Arterial line monitoring	86 (51.2)
Greatest MAP reduction (mmHg), mean ± SD	41.7 ± 17.9
Duration of MAP reduction (min), median (IQR)	
≥10 mmHg	10 (5,25)
≥15 mmHg	10 (5,20)
≥20 mmHg	15 (8.8,30)
≥25 mmHg	15 (10,30)
≥30 mmHg	70 (30,110)
Vasopressor use	103 (62)
Ephedrine (mg), median (IQR)	6 (0,10.5)
Levophed (mcg), median (IQR)	15 (0,145)
**Procedural outcomes, complications, and follow-up**	
Time from groin puncture to recanalization (min), median (IQR)	64.5 (38.2,99.5)
Post-procedural 24 h MAP (mmHg), mean ± SD	92.2 ± 11
Post-procedural 24 h NIHSS	
1–4	33 (19.9)
5–15	107 (64.5)
16–20	13 (7.8)
21–42	13 (7.8)
Reperfusion grade (mTICI)	
0	6 (3.6)
1	2 (1.2)
2a	20 (12)
2b	45 (27.1)
2c	3 (1.8)
3	90 (54.2)
Complications	
Groin hematoma	10 (6)
Cerebral vasospasm	1 (0.6)
Vessel perforation	1 (0.6)

Hemorrhagic transformation	65 (39.2)
Ventilator days (days), median (IQR)	4 (2,7)
Hospital stay (days), median (IQR)	9 (5,18.8)
Discharge status	
Fully recovery	11 (6.6)
Disability	82 (49.4)
Bedridden	51 (30.7)
Death	22 (13.3)
mRS at 90 days	
0–2	72 (43.4)
3–6	94 (56.6)
Mortality at 90 days	40 (24.1)

SD, standard deviation; BMI, body mass index; NIHSS, National Institutes of Health Stroke Scale; MAP, mean arterial pressure; IQR, interquartile range; mTICI, modified Thrombolysis in Cerebral Infarction.

Supplementary Table S2 Clinical, anesthetic, and procedural factors according to outcome and mortality at 90 days

**Variable**	**Good outcome (mRS 0–2)** (n = 72)	**Poor outcome (mRS 3–6)** (n = 94)	**P-value**	**Survivors** (n = 126)	**Nonsurvivors** (n = 40)	***P*-value**
Age (years), median (IQR)	64.5 (55.8,74)	75.5 (63.2,80.8)	<0.001	70.5 (61,78)	74.5 (57,78.2)	0.813
Sex			0.06			0.530
Male	51 (70.8)	52 (55.3)		76 (60.3)	27 (67.5)	
Female	21 (29.2)	42 (44.7)		50 (39.7)	13 (32.5)	
BMI (kg/m^2), median (IQR)	23.4 (21.5,25.8)	23.6 (20.8,26)	0.794	23.5 (21.3,25.9)	23.4 (20.3,25.7)	0.565
Comorbidities						
Diabetes mellitus	10 (13.9)	33 (35.1)	0.004	27 (21.4)	16 (40)	0.033
Hypertension	40 (55.6)	63 (67)	0.178	78 (61.9)	25 (62.5)	1
Dyslipidemia	34 (47.2)	43 (45.7)	0.974	65 (51.6)	12 (30)	0.028
Prior stroke	18 (25)	22 (23.4)	0.956	28 (22.2)	12 (30)	0.430
Atrial fibrillation	27 (37.5)	46 (48.9)	0.189	58 (46)	15 (37.5)	0.445
CAD	12 (16.7)	21 (22.3)	0.477	25 (19.8)	8 (20)	1
VHD	13 (18.1)	13 (13.8)	0.598	18 (14.3)	8 (20)	0.537
Malignancy	6 (8.3)	10 (10.6)	0.815	12 (9.5)	4 (10)	1
Smoking	16 (22.2)	14 (14.9)	0.311	23 (18.3)	7 (17.5)	1
Occlusion location			0.501			0.379
ICA-T	23 (31.9)	32 (34)		42 (33.3)	13 (32.5)	
M1 segment	34 (47.2)	37 (39.4)		37 (45.2)	14 (35)	
M2 segment	8 (11.1)	7 (7.4)		12 (9.5)	3 (7.5)	
Vertebral artery	1 (1.4)	2 (2.1)		2 (1.6)	1 (1.4)	
Basilar artery	5 (6.9)	13 (13.8)		10 (7.9)	8 (20)	
ACA	0 (0)	2 (2.1)		1 (0.8)	1 (2.5)	
A2 segment	1 (1.4)	0 (0)		1 (0.8)	0 (0)	
Carotid artery	0 (0)	1 (1.1)		1 (0.8)	0 (0)	
Preoperative NIHSS						
Mean ± SD	12.9 ± 4.8	15.5 ± 4.5	<0.001	13.8 ± 4.6	16.1 ± 4.8	0.008
1–4	3 (4.2)	1 (1.1)	0.059	4 (3.2)	0 (0)	0.091
5–15	48 (66.7)	49 (52.1)		77 (61.1)	20 (50)	
16–20	18 (25)	33 (35.1)		38 (30.2)	13 (32.5)	
21–42	3 (4.2)	11 (11.7)		7 (5.6)	7 (17.5)	
Baseline MAP (mmHg), mean ± SD	108.9 ± 18	110.4 ± 19.9	0.616	110.4 ± 18.7	107.8 ± 20.2	0.447
Prior treatment with IV thrombolysis	39 (54.2)	44 (46.8)	0.434	64 (50.8)	19 (47.5)	0.856
Time from last-known-well to groin puncture (min), median (IQR)	291.5 (190,414.2)	289 (211.2,408.8)	0.748	291.5 (205,422.5)	284 (230,397.5)	0.998
Time from onset to groin puncture (min), median (IQR)	230 (169,295)	244 (185.5,373.8)	0.267	230 (173.5,363.8)	250 (191.2,365.5)	0.344

Preoperative laboratory						
Hematocrit (%), median (IQR)	40.3 (36.7,43.2)	39.1 (35.1,41.9)	0.116	39.9 (36.2,42.6)	37 (34.6,41.9)	0.111
Platelet (cell/mm^3), median (IQR)	231000 (184000,276750)	212500 (178250,269750)	0.224	224000 (180000,272750)	214500 (177250,276000)	0.740
Glucose (mg/dL)	124	137	0.003			<0.001
- Median (IQR)	(105.5,139.5)	(117,179.8)		125.5 (107,149.8)	155.5 (123.5,191.5)	
- <140	54 (75)	49 (52.1)	0.004	87 (69)	16 (40)	0.002
- ≥140	18 (25)	45 (47.9)		39 (31)	24 (60)	
Creatinine (mg/dL), median (IQR)	1 (0.7,1.2)	1 (0.7,1.3)	0.951	1 (0.74,1.2)	1 (0.6,1.5)	0.878
Induction agents			<0.001			<0.001
Propofol	61 (84.7)	50 (53.2)		94 (74.6)	17 (42.5)	
Etomidate	1 (1.4)	1 (1.1)		2 (1.6)	0 (0)	
Midazolam	3 (4.2)	12 (12.8)		6 (4.8)	9 (22.5)	
≥2 agents	3 (4.2)	26 (27.7)		20 (15.9)	9 (22.5)	
None	4 (5.6)	5 (5.3)		4 (3.2)	5 (12.5)	
Induction dose			<0.001			<0.232
Optimal dose	35 (48.1)	21 (23.6)		47 (38.5)	9 (25.7)	
Non-optimal dose	33 (45.8)	68 (76.4)		75 (61.5)	26 (74.3)	
Intubation location			0.017			<0.001
Referring hospital	7 (9.7)	18 (19.1)		9 (7.1)	16 (40)	
Emergency room	1 (1.4)	9 (9.6)		6 (4.8)	4 (10)	
Ward	1 (1.4)	64 (68.1)		3 (2.4)	1 (2.5)	
Interventional suite	63 (87.5)	3 (3.2)		108 (85.7)	19 (47.5)	
Intubation attempt			0.259			0.144
1 attempt	72 (100)	91 (96.8)		125 (99.2)	38 (95)	
≥2 attempts	0 (0)	3 (3.2)		1 (0.8)	2 (5)	
Induction duration (min), mean (IQR)	15 (10,18.5)	15 (10.20)	0.848	15 (10.18.5)	15 (10,20)	0.908
Maintenance of anesthesia			0.379			1
Sevoflurane	68 (94.4)	92 (97.9)		121 (96)	39 (97.5)	
Desflurane	3 (4.2)	2 (2.1)		4 (3.2)	1 (2.5)	
Propofol infusion	1 (1.4)	1 (0)		1 (0.8)	0 (0)	
Arterial line monitoring	31 (43.1)	54 (57.4)	0.093	64 (50.8)	17 (42.5)	0.464
Greatest MAP reduction (mmHg), mean ± SD	40.4 ± 17.1	42.7 ± 18.5	0.416	42.7 ± 17.8	38.7 ± 17.9	0.220
Duration of MAP reduction (min), median (IQR)	5 (0,10)	5 (0,15)	0.852	10 (5,25)	15 (5,17.5)	0.958
≥10 mmHg	5 (0,15)	0 (0,10)	0.147	10 (5,20)	15 (6.2,20)	0.405
≥15 mmHg	5 (0,15)	5 (0,15)	0.770	15 (5,35)	12.5 (5,21.2)	0.363
≥20 mmHg	7.5 (0,20)	10 (0,20)	0.857	10 (10,25)	20 (10,30)	0.452
≥25 mmHg	42.5 (0,72.5)	50 (5,115)	0.132	65 (27.5,100)	100 (50,123.8)	0.073
≥30 mmHg						
Vasopressor use	36 (50)	67 (71.3)	0.008	77 (61.1)	26 (65)	0.799
Ephedrine (mg), median (IQR)	6 (2.2,12)	0 (0,6)	0.006	6 (0,12)	0 (0,6)	0.157
Levophed (mcg), median (IQR)	2.5(0,22.5)	20 (0,320)	0.008	5 (0,60)	65 (12.5,463.8)	0.003
Time from groin puncture to recanalization (min), median (IQR)	55 (37.5,85)	70.5 (39.2,111)	0.078	61 (38,88.2)	75 (48.8,114.8)	0.089

Extubation after MT	71 (98.6)	56 (59.6)	<0.001	118 (93.7)	9 (22.5)	<0.001
Post-procedural 24 h MAP (mmHg), mean ± SD/ median (IQR)	91.2 ± 9.2	93 ± 12.1	0.302	90.5 (85,97)	94.5 (82,99)	0.722
Post-procedural 24 h NIHSS, median (IQR)/	5.5 (3,8)	12 (8,16)	<0.001	8.2 ± 4.9	15.3 ± 6.5	<0.001
mean ± SD	30 (41.7)	33 (19.9)	<0.001	32 (25.4)	1 (2.5)	<0.001
1–4	41 (56.9)	107 (64.5)		86 (68.3)	21 (52.5)	
5–15	1 (1.4)	13 (7.8)		6 (4.8)	7 (17.5)	
16–20	0 (0)	13 (7.8)		2 (1.6)	11 (27.5)	
21–42						
Reperfusion grade			0.018			0.021
(mTICI)	6 (8.3)	22 (23.4)		16 (12.7)	12 (30)	
0, 1, 2a	66 (91.7)	72 (76.6)		110 (87.3)	28 (70)	
2b, 2c, 3						
Complications						
Groin hematoma	5 (6.9)	5 (5.3)	0.748	9 (7.1)	1 (2.5)	0.454
Cerebral vasospasm	0 (0)	1 (1.1)	1	1 (0.8)	0 (0)	1
Vessel perforation	0 (0)	1 (1.1)	1	1 (0.8)	0 (0)	1
Hemorrhagic transformation	17 (23.6)	48 (51.1)	<0.001	45 (35.7)	20 (50)	0.154
Ventilator days (days), median (IQR)	2 (1,3)	5 (3,12)	<0.001	3 (2,7)	4 (3,7.8)	0.014
Hospital stay (days), median (IQR)	8 (5,16)	12 (5,20.8)	0.192	10.5 (6,20.8)	5 (4,14.5)	<0.001
Discharge status			<0.001			<0.001
Fully recovery	11 (6.6)	0 (0)		11 (8.7)	0 (0)	
Disability	60 (83.3)	22 (23.4)		81 (64.3)	1 (2.5)	
Bedridden	1 (1.4)	50 (53.2)		34 (27)	17 (42.5)	
Death	0 (0)	22 (23.4)		0 (0)	22 (55)	
mRS at 90 days			<0.001			<0.001
0–2	72 (100)	1 (1.1)		73 (57.9)	0 (0)	
3–6	0 (0)	93 (98.9)		53 (42.1)	40 (100)	
Mortality at 90 days	0 (0)	40 (42.6)	<0.001			

mRS, modified Rankin Scale; SD, standard deviation; BMI, body mass index; NIHSS, National Institutes of Health Stroke Scale; MAP, mean arterial pressure; IQR, interquartile range; mTICI, modified Thrombolysis in Cerebral Infarction; CAD, ; VHD, ; ICA-T, ; ACA .

Supplementary Table S3 Multivariable logistic regression analysis for poor functional outcome (mRS 3–6) at 90 days after exclusion of post-procedural variables

**Variable**	**Adjusted Odds Ratio**	**95% CI**	***P*-value**
Preoperative glucose ≥ 140 mg/dL	3.20	1.42–7.59	0.006
Optimal induction dose	0.20	0.08–0.46	<0.001
Vasopressor use	4.24	1.89–10.08	<0.001
Reperfusion grade (mTICI)	6.70	2.03–26.82	0.003

mTICI, modified Thrombolysis in Cerebral Infarction; CI, confidence interval

Supplementary Table S4 Multivariable logistic regression analysis for 90-day mortality after exclusion of post-procedural variables

**Variable**	**Adjusted Odds Ratio**	**95% CI**	***P*-value**
Hematocrit	0.94	0.94–1.00	0.047
Preoperative glucose ≥ 140 mg/dL	3.01	1.39–6.64	0.005
Vasopressor use	1.0014	1.0001-1.0028	0.037
Reperfusion grade (mTICI)	6.86	1.69–31.72	0.009

mTICI, modified Thrombolysis in Cerebral Infarction; CI, confidence interval

Supplementary Figure S1 Receiver operating characteristic curve of the sensitivity model for predicting poor functional outcome at 90 days after exclusion of post-procedural variables.

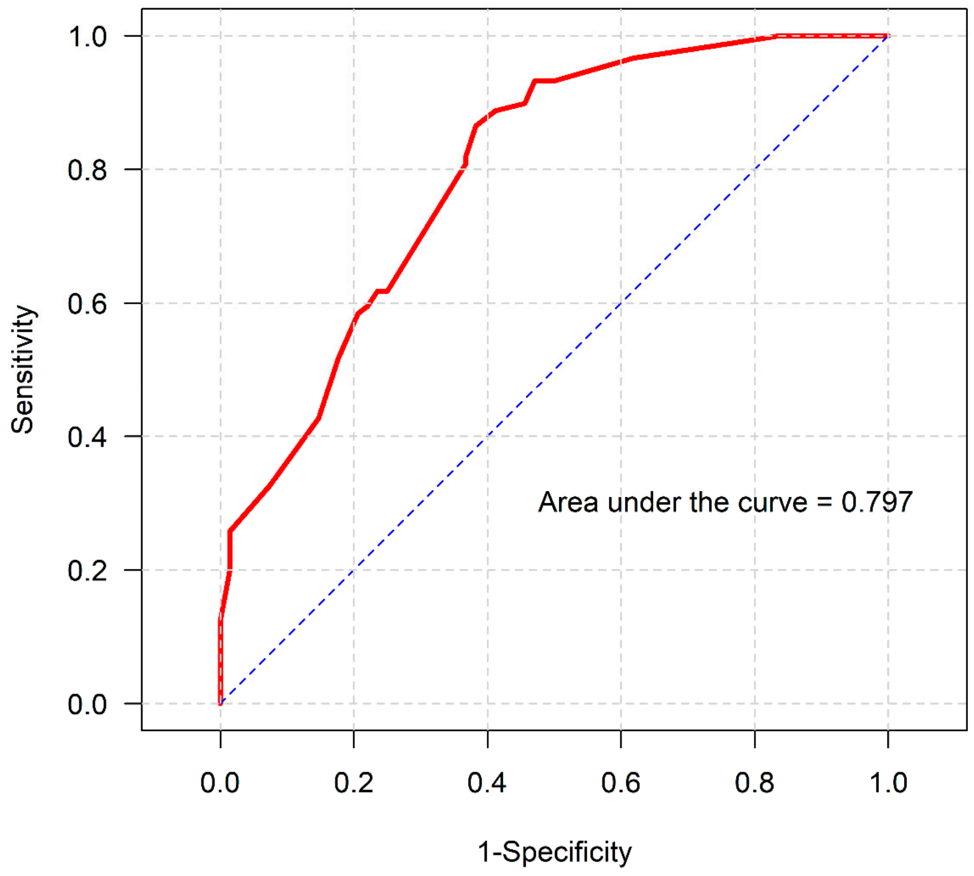

Supplementary Figure S2 Receiver operating characteristic curve of the sensitivity model for predicting 90-day mortality after exclusion of post-procedural variables.

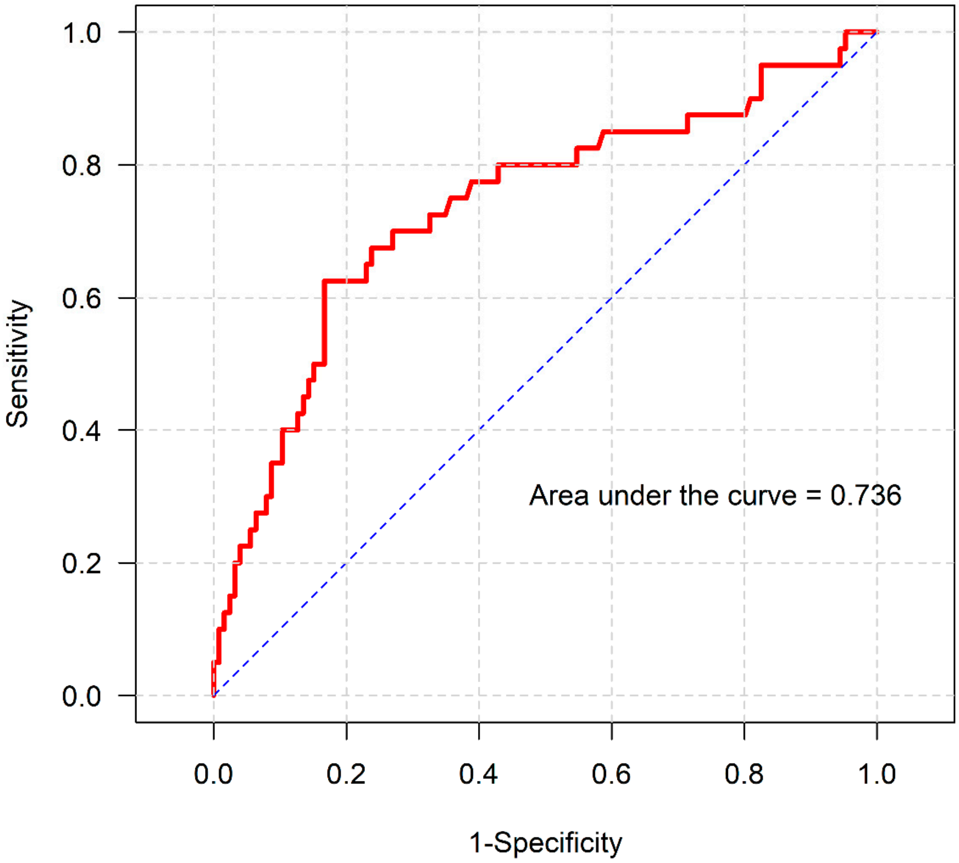

Supplementary Figure S3 Calibration plot for the multivariable model predicting poor functional outcome at 90 days.

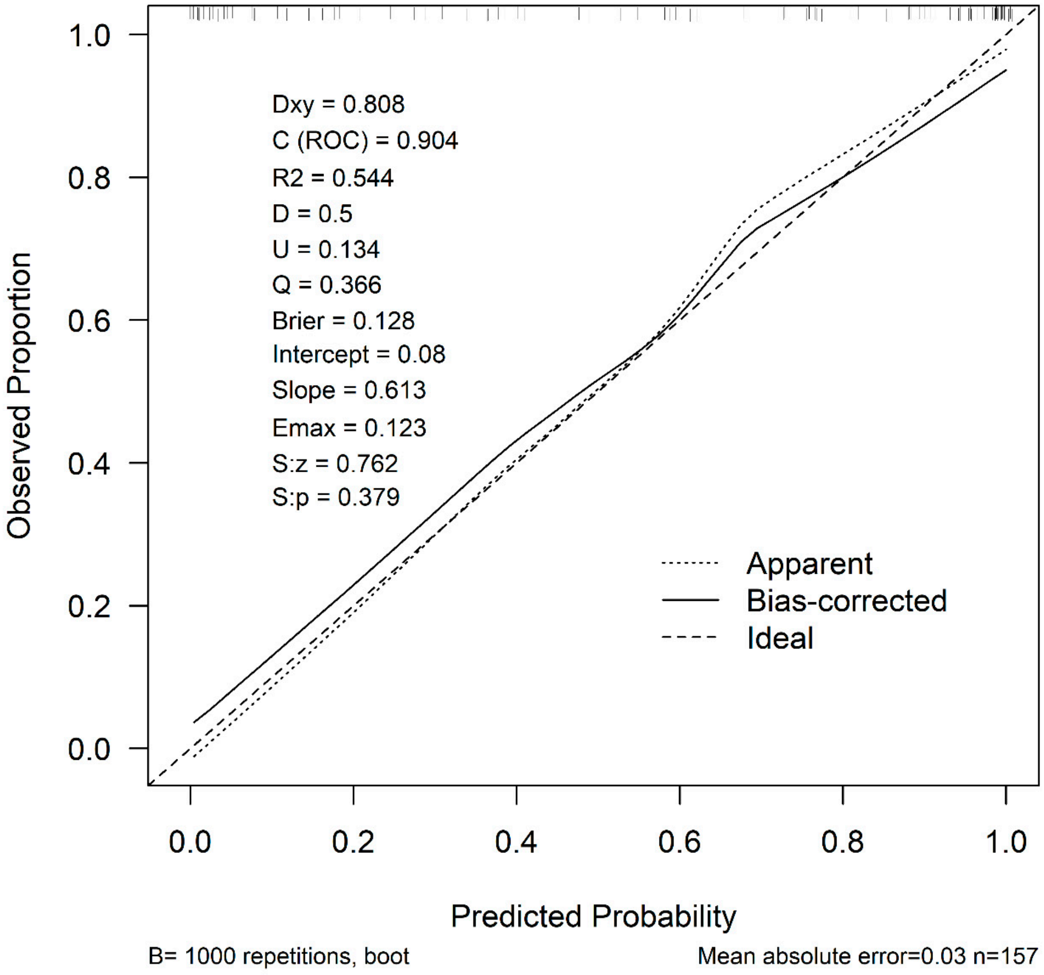

Supplementary Figure S4 Calibration plot for the multivariable model predicting 90-day mortality.

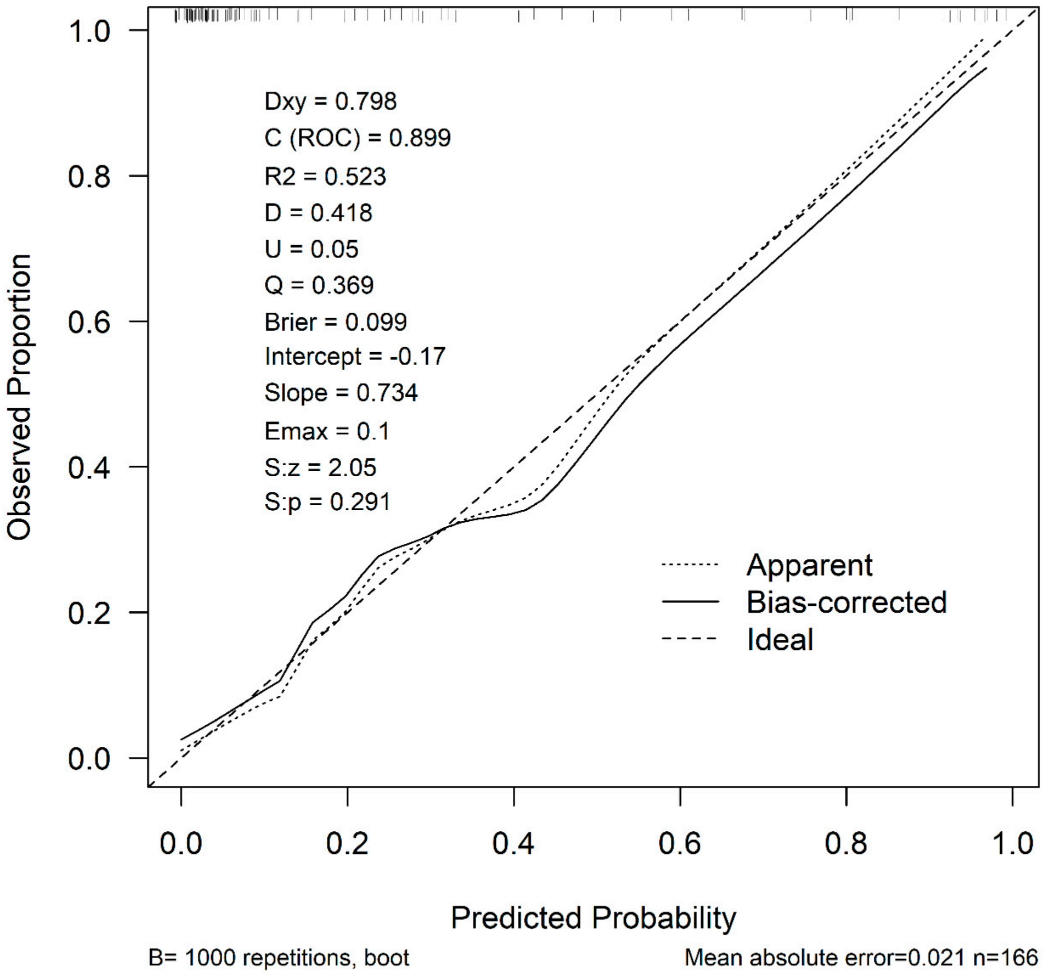

Supplementary Figure S5 Calibration plot of the sensitivity model for predicting poor functional outcome (mRS 3–6) at 90 days after exclusion of post-procedural variables.

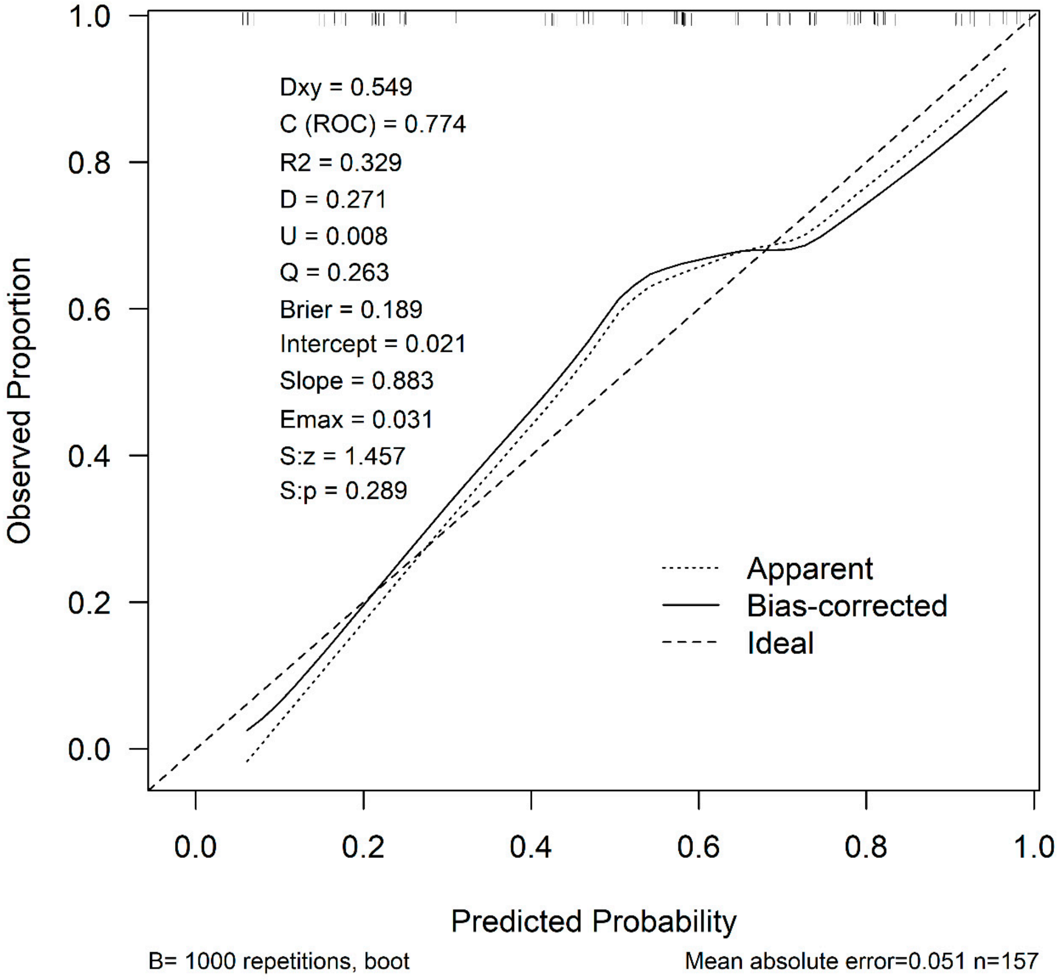

Supplementary Figure S6 Calibration plot of the sensitivity model for predicting 90-day mortality after exclusion of post-procedural variables.

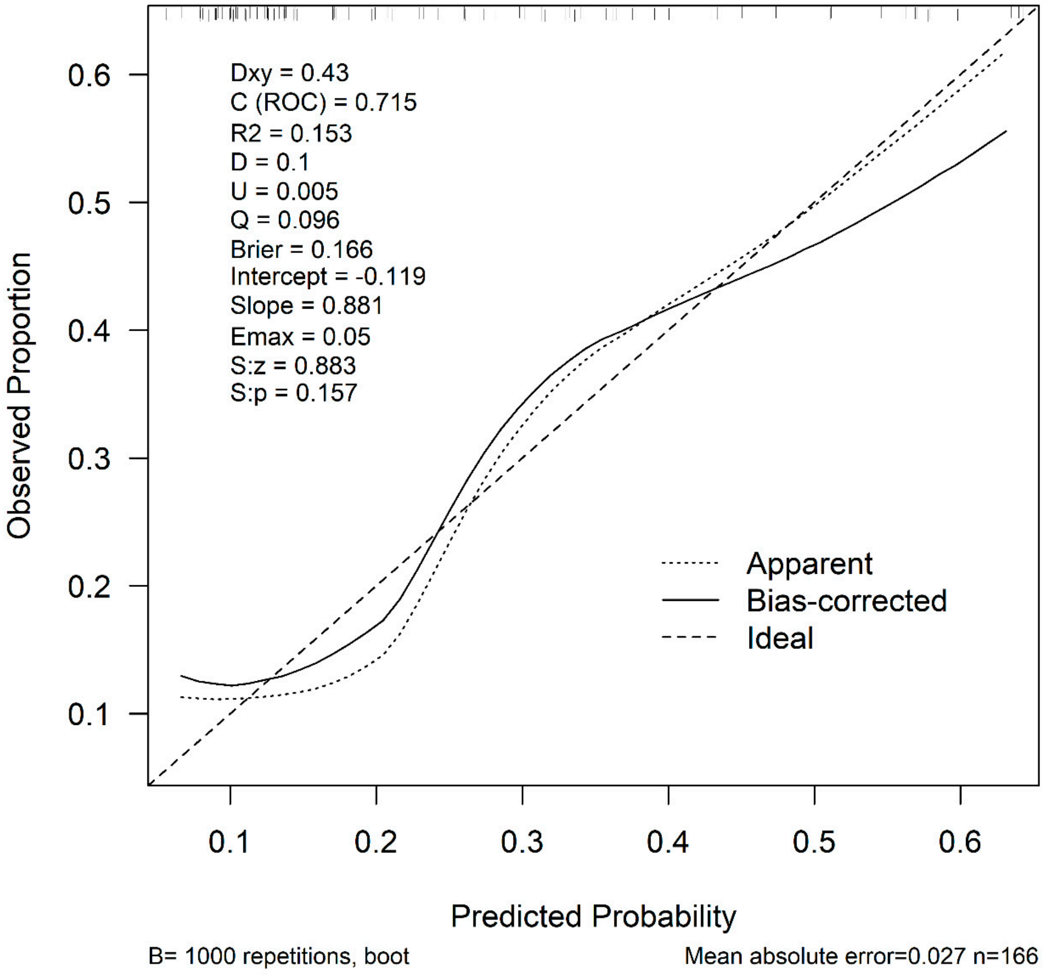

Supplement: Supplementary file 1 [file jcm-15-03332-s001.zip › jcm-4237170-supplementary.pdf]
